# Five decades of clinical assessment of whole-sporozoite malaria vaccines

**DOI:** 10.3389/fimmu.2022.977472

**Published:** 2022-09-08

**Authors:** Helena Nunes-Cabaço, Diana Moita, Miguel Prudêncio

**Affiliations:** Instituto de Medicina Molecular João Lobo Antunes, Faculdade de Medicina, Universidade de Lisboa, Lisboa, Portugal

**Keywords:** vaccine, plasmodium, sporozoite, clinical trial, protective efficacy, immunogenicity

## Abstract

In 1967, pioneering work by Ruth Nussenzweig demonstrated for the first time that irradiated sporozoites of the rodent malaria parasite *Plasmodium berghei* protected mice against a challenge with infectious parasites of the same species. This remarkable finding opened up entirely new prospects of effective vaccination against malaria using attenuated sporozoites as immunization agents. The potential for whole-sporozoite-based immunization in humans was established in a clinical study in 1973, when a volunteer exposed to X-irradiated *P. falciparum* sporozoites was found to be protected against malaria following challenge with a homologous strain of this parasite. Nearly five decades later, much has been achieved in the field of whole-sporozoite malaria vaccination, and multiple reports on the clinical evaluation of such candidates have emerged. However, this process has known different paces before and after the turn of the century. While only a few clinical studies were published in the 1970’s, 1980’s and 1990’s, remarkable progress was made in the 2000’s and beyond. This article reviews the history of the clinical assessment of whole-sporozoite malaria vaccines over the last forty-nine years, highlighting the impressive achievements made over the last few years, and discussing some of the challenges ahead.

## Introduction

The 6 October 2021 will be forever engraved in the history of the fight against malaria as the date when RTS,S, the first vaccine against this devastating disease, was recommended by the World Health Organization (WHO) to be given to children living in regions with moderate-to-high transmission of *Plasmodium falciparum* (Pf) malaria. RTS,S, a subunit vaccine based on the Pf circumsporozoite protein (CSP), was initially developed by the Walter Reed Army Institute of Research (WRAIR) and GlaxoSmithKline (GSK), in 1987. A long path followed, during which the vaccine was evaluated in multiple clinical trials in malaria-endemic regions, leading to its eventual endorsement. Immunogenicity studies have indicated that RTS,S exerts its protective effect through antibodies against PfCSP and through CD4^+^ T cell responses, but no clear immune correlates of protection have been identified (1, 2). Results from a large Phase III clinical study have shown that 4 doses of the vaccine present relatively modest and rapidly waning 25.9% and 17.3% effectiveness against clinical and severe malaria, respectively, in newborns aged 6–12 weeks, and 36.3% and 32.2% efficiency against clinical and severe malaria, respectively, in children aged 5–17 months [(3) and reviewed in (4)]. A post-approval plan comprising 4 complementary Phase IV studies that will evaluate safety, effectiveness and impact of RTS,S in the context of its real-life implementation will support the ongoing evaluation of the vaccine’s benefit-risk and inform decision-making for its potential wider implementation across sub-Saharan Africa (5). Moreover, RTS,S is not expected to protect against the other human malaria parasites, namely *P. vivax* (Pv), *P. ovale*, *P. malariae*, and the zoonotic *P. knowlesi* (6). Thus, in spite of this landmark achievement, the licensing of RTS,S should not be viewed as the end of the road in the quest for a malaria vaccine. Rather, it should be seen as a stepping stone towards the WHO’s ambitious goals of, by 2030, licensing vaccines targeting Pf and Pv with protective efficacy of at least 75 percent against clinical malaria and that substantially reduce the incidence of human malaria infection (7).

**Figure 1:**
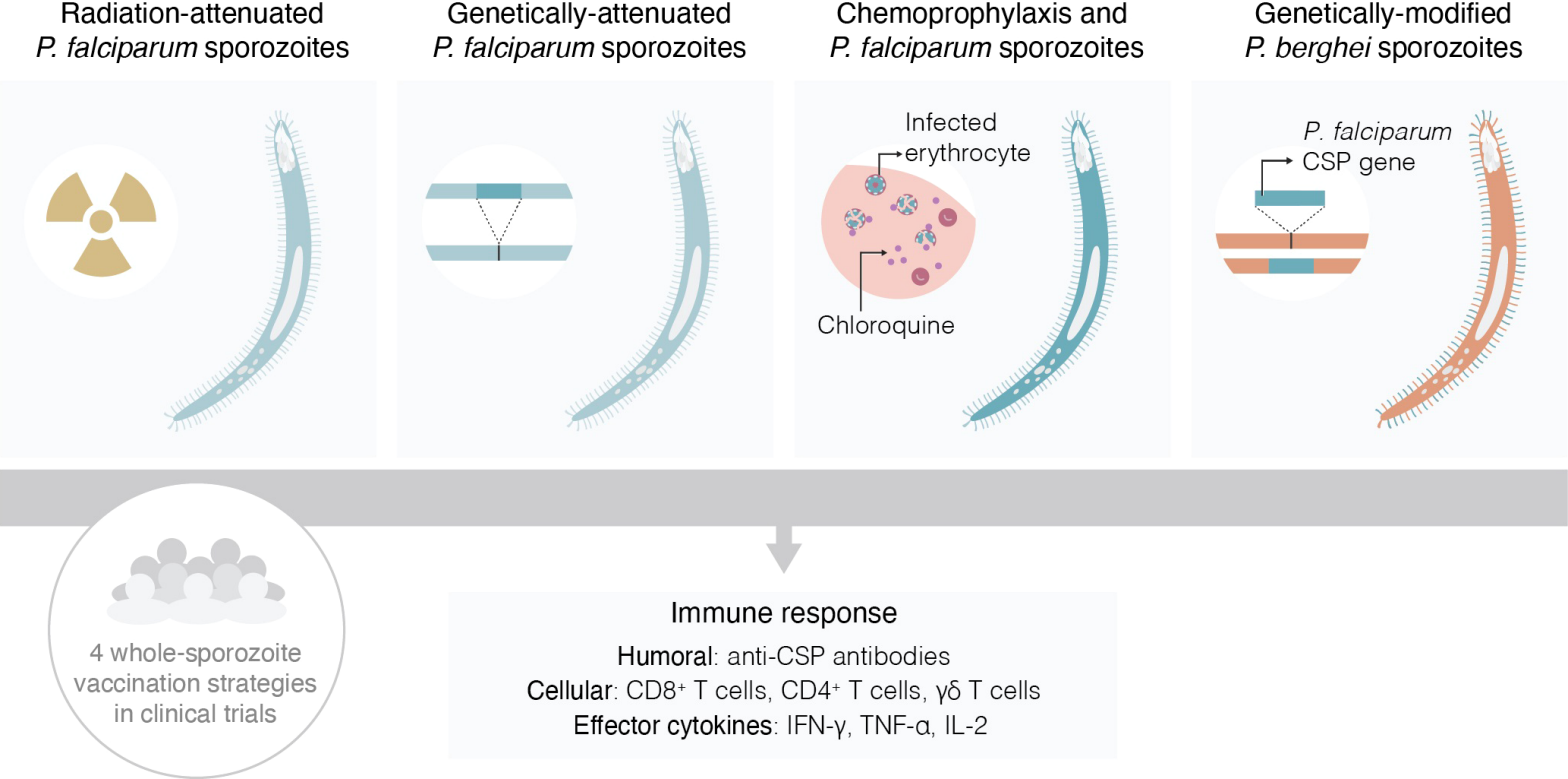
Schematic representation of the four types of whole-sporozoite vaccines against malaria assessed in clinical trials.

Whole-sporozoite (WSp) vaccines (**Figure 1**) have emerged as a possible strategy to immunize against malaria since the demonstration that X-irradiated sporozoites of *P. berghei* (Pb) could induce protective immune responses against an intravenous challenge with fully infective Pb parasites (8). Interest in WSp vaccination increased following the initial demonstration by Clyde *et al.* that radiation-attenuated Pf sporozoites could also afford protective immunity against homologous Pf malaria (9). However, for a long time, WSp vaccination was considered impractical, and the barriers to the development of WSp vaccines seemed all but insurmountable (10). Nevertheless, research into this area gained momentum in the early 2000’s and, one by one, many of these barriers were overcome, through the efforts of several laboratories around the world and, pivotally, by the remarkable technological and scientific progress made by Stephen L. Hoffman’s team at Sanaria, Inc. and its network of collaborators.

**Figure 2:**
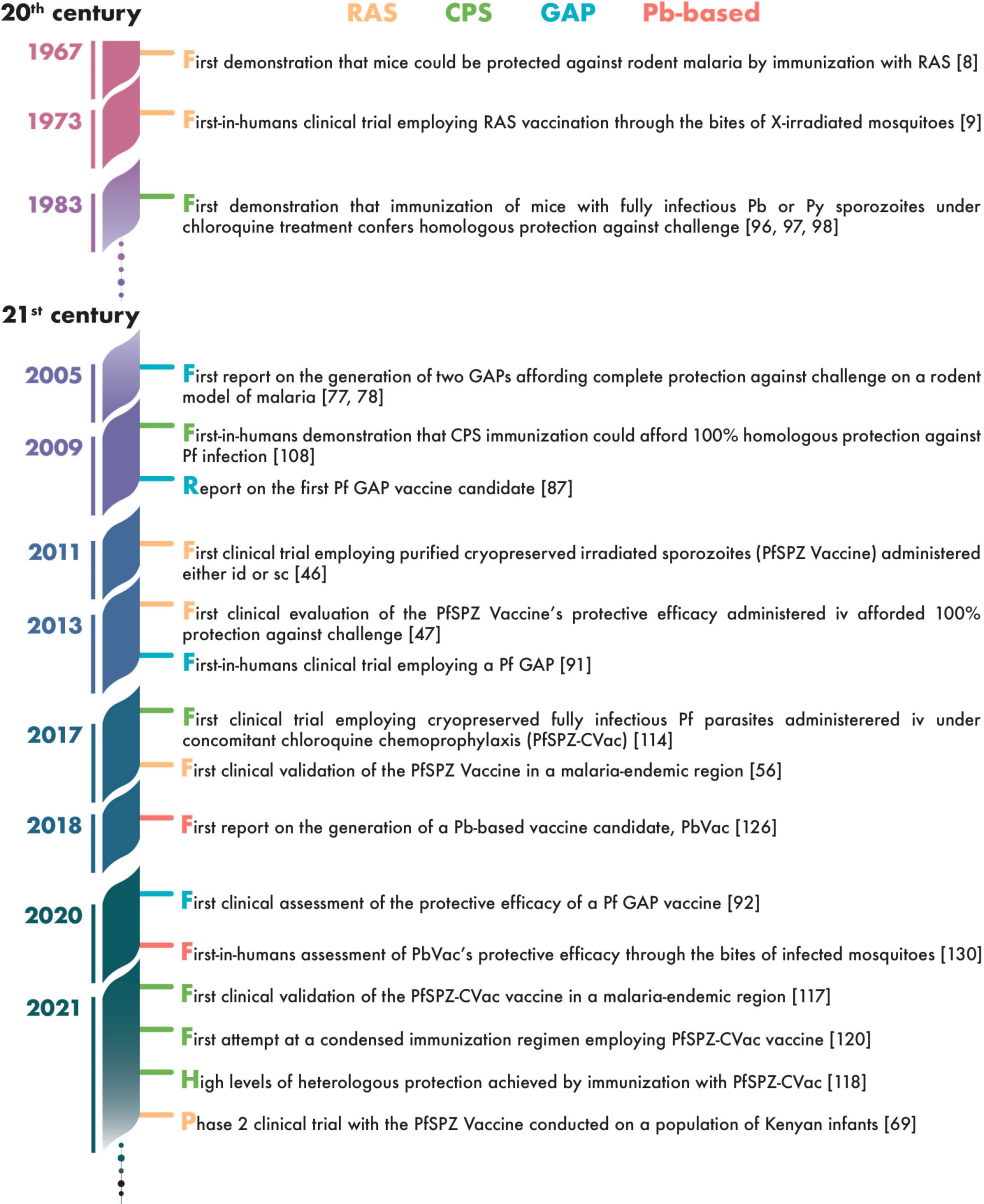
Timeline of landmark achievements in the development of whole-sporozoite vaccines against malaria.

Nearly five decades have elapsed since the first clinical assessment of a WSp vaccine by Clyde *et al.*, in 1973 (9). Whereas throughout the first 3 decades of this period such trials involved a total of only about two dozen human subjects (10, 11), this number has risen exponentially since then, generating an impressive amount of data on the immunogenicity and protective efficacy of WSp vaccines in humans (**Figure 2**). Here, we review the knowledge accumulated through these clinical studies, at a time when the prospect of WSp vaccines becoming a reality in a not-so-distant future seems more realistic than ever.

## Clinical evaluation of whole-sporozoite vaccines

### Controlled human malaria infection

The widely used term Controlled Human Malaria Infection (CHMI) is technically incorrect, since, as McFadden eloquently states, “malaria is a disease, not an organism” (12). As such, describing infection by *Plasmodium* parasites as “malaria infections” is no more right than referring to HIV infections as “AIDS infections” or to SARS-CoV-2 infections as “COVID-19 infections”. However, the term CHMI appears to have been adopted by the community and, since it seems unlikely that it will be replaced by the more accurate “Controlled Human Infection by Malaria Parasites” (CHIMP) or “Controlled Human *Plasmodium* Infection” (CHPI), will be employed throughout this review.

CHMI is of paramount importance in the context of malaria vaccinology, as amply discussed in several reviews (13–20). Both early and recent studies aimed at assessing WSp vaccine candidates in the clinic have resorted to CHMI, employing the strictly controlled exposure of trial participants to the bites of laboratory-reared, *Plasmodium*-infected mosquitoes (21). CHMI by the bites of five mosquitoes consistently infects all malaria-naïve volunteers (22), although exposure to the bites of 3 aseptically-raised Pf-infected mosquitoes has also been proposed as a safe, effective procedure for CHMI in malaria-naïve adults (23). While the NF54 strain of Pf is most commonly employed for CHMI by mosquito bite, the 7G8, NF135.C10 and NF166.C8 Pf strains have also been reported as eligible for use in such studies (24). An alternative to mosquito bite-based CHMI lies in the use of Sanaria, Inc.’s PfSPZ Challenge, consisting of infectious, aseptic, purified, vialed, cryopreserved Pf sporozoites, which can be administered by needle and syringe (25). Dose-finding trials have shown that intravenous (iv) injection of 3200 PfSPZ Challenge leads to a geometric mean pre-patent period similar to that observed following the bites of 5 Pf-infected mosquitoes (26). Whether CHMI by mosquito bite is preferable to the iv route, or vice-versa, remains a matter of some controversy. While the former is the more natural route of infection, it does not allow the exact estimation of the number of inoculated sporozoites. Nevertheless, efforts have been made to standardize mosquito-administered CHMI (27), reducing the impact of this biological variability. On the other hand, PfSPZ Challenge enhances access to CHMI, including in malaria-endemic regions [(28, 29) and recently reviewed in (30)], which otherwise would be limited to the few research facilities with the capability to carry out Pf infections of mosquitoes for experimental purposes (25).

## Radiation-attenuated sporozoites

An appropriate dose of ionizing radiation (UV, X-ray and γ) can prevent replication of a pathogenic organism, while preserving metabolic activity (31). Radiation-attenuated *Plasmodium* sporozoites (RAS) retain their ability to infect liver cells but are unable to replicate and progress to form erythrocyte-infectious merozoites, likely as a result of extensive DNA damage, accompanied by downregulation of DNA repair genes (32). In 1967, Ruth Nussenzweig and colleagues reported for the first time that mice could be protected against rodent malaria by immunization with RAS (8). Publication of this report created hope that humans could be completely protected against malaria, inspiring others to explore the prospect of WSp immunization in the clinic (33). To this day, RAS remain the gold-standard of whole-organism vaccination against human malaria.

### Early studies of RAS immunization in humans

Inspired by Ruth Nussenzweig’s pioneering report, in 1973, a team at the University of Maryland School of Medicine commenced trials to vaccinate human volunteers with Pf RAS, delivered by the bites of X-irradiated mosquitoes. In the first report of these studies, one of three volunteers fed on by 379 mosquitoes over the course of 84 days did not develop malaria following an infective challenge with sporozoites delivered by non-irradiated mosquitoes 15 days after the last immunization (9). This volunteer then underwent an additional 5 immunization sessions, during which he was exposed to a total of 819 irradiated mosquitoes, and remained protected against a second infectious sporozoite challenge 12 days after the last immunization (9). These observations constitute the first demonstration of the protective efficacy of RAS vaccination in the clinic. Interestingly, having remained malaria-free for 2 months after the second sporozoite challenge, the same volunteer was challenged by intravenous injection of Pf trophozoites, and developed parasitemia and clinical symptoms 5 days later (9). Although the authors may not have fully realized this at the time, this was also the first indication that the protection afforded by WSp vaccination is purely restricted to the pre-erythrocytic stage of *Plasmodium* infection. A subsequent report describes the first RAS immunization against both Pf and Pv through the bites of irradiated mosquitoes infected with either of these parasites. A single volunteer received immunizing doses of Pf or Pv on different days and at different intervals, and was subsequently challenged with infectious parasites of either species delivered by non-irradiated mosquitoes. The experimental setup employed might be considered less than appropriate nowadays, particularly considering the small number of study participants, the irregular immunization schedules, and the concomitant use of both human parasites. The subject underwent immunization with Pf sporozoites delivered by a total of 1806 irradiated mosquitoes, which protected him against a Pf but not a Pv challenge. Subsequent immunization by exposure to a total of 739 Pv-infected, irradiated mosquitoes conferred protection against Pv challenges for up to six months (34). Another volunteer immunized by the bites of 728 irradiated Pv-infected mosquitoes was reported to be unprotected against a Pv challenge one week after the last immunization, but was protected one week after the last inoculation of an additional series of 1251 bites (35). Finally, three volunteers immunized by the bites of 440-987 irradiated, Pf-infected mosquitoes were protected for 8 weeks against an infectious Pf challenge, but no protection was observed in volunteers exposed to 200 or fewer irradiated mosquitoes (36). Overall, of 11 volunteers who were immunized in the 1970s by the bites of irradiated, Pf-infected mosquitoes, five displayed species-specific (37) protection against a subsequent exposure to infective sporozoites of different Pf strains.

It would be more than a decade until the next clinical studies of WSp vaccines took place. In 1991, two groups of volunteers were vaccinated by repeated exposure to the bites of Pf-infected, X-irradiated mosquitoes. While two volunteers in group 1, exposed to 625 and 715 irradiated mosquitoes, were unprotected against an infectious Pf challenge delivered by mosquito bite, all three volunteers in group 2, who were exposed to a total of 1563-1681 immunizing bites, were fully protected against a Pf challenge three weeks after the last immunization (38). One of these subjects received a series of booster immunization bites approximately three months after that first challenge and was re-challenged nine months after that, remaining immune to virulent sporozoites (39). Between 1989 and 1999, another eleven volunteers were immunized at the Naval Medical Research Center and the Walter Reed Army Institute for Research. The results of these trials are summarized in a publication by Hoffman *et al.* in 2002, and show that ten of eleven volunteers immunized by the bites of 1001-2927 irradiated mosquitoes infected with Pf strain NF54 were protected against a homologous challenge two to nine weeks after the last immunization (11). Furthermore, four out of five protected subjects were also protected against a Pf re-challenge 23-42 weeks after a secondary immunization, and two volunteers were protected when re-challenged with the heterologous 7G8 strain of Pf (11). This report constitutes a landmark in WSp malaria vaccination, demonstrating not only that protective immunity elicited by Pf RAS is strain-transcendent, but also that it may persist for at least 10 months. These findings created a renewed interest in WSp vaccination against human malaria and paved the way for an entirely new era of research in this field.

### WSp vaccination by injection: purification and cryopreservation of Pf sporozoites

The enthusiasm generated by the observations outlined above was curbed by the generally accepted conviction that a vaccine whose administration required the bites of more than 1000 mosquitoes was clinically impractical [reviewed in (40, 41)]. However, and contrary to what had successfully been done in rodent models, the injection of infected mosquito salivary gland material into humans posed unacceptable medical risks (41). This realization entailed several immediate concerns, arising from the (i) practical limitations in infecting mosquitoes with Pf, which depended on feeding on volunteers with circulating Pf gametocytes; (ii) relatively small numbers of sporozoites in the salivary glands of Pf-infected mosquitoes; and (iii) absolute necessity for adequate purification and preservation of Pf sporozoites intended for immunization. The first of these concerns had been overcome by the development of methods for *in vitro* culturing of Pf parasites in 1976 (42), and for gametocyte production from these cultures in 1982 (43). The challenges imposed by the other two concerns meant that, for the best part of the first decade of the 21^st^ century, clinical trials employing RAS remained scarce (44). This situation changed dramatically thanks to the persistence of Stephen L. Hoffman and his team at Sanaria, Inc., who set out to develop methods to increase sporozoite yields in infected mosquitoes, as well as to purify and preserve these parasites (40). Their efforts culminated in the successful manufacture of the PfSPZ Vaccine, consisting of aseptically purified, metabolically active, non-replicating (irradiated), cryopreserved Pf sporozoites of the NF54 strain, suitable for clinical use (45) and GMP-compliant (31). This remarkable achievement completely changed the prospects for WSp vaccination, and prompted a surge of clinical trials to assess and optimize the immunogenicity and efficacy of RAS-based immunization.

### Establishing the proof-of-principle of PfSPZ vaccination

In the first attempt at human vaccination with PfSPZ Vaccine, the vaccine was administered either by intradermal (id) or subcutaneous (sc) injection to a total of 80 volunteers, 44 of whom subsequently underwent homologous CHMI by mosquito bite, alongside 18 non-immunized controls. The results were nothing less than disappointing, with only two of the challenged vaccinees protected against infection, and none of the others displaying even a delay in time to detectable parasitemia (46). Unfazed by these results, the authors employed several animal models to dissect the immune responses elicited by injection of the vaccine through different routes. Their results provided unequivocal evidence that intravenous (iv) injection of PfSPZ Vaccine elicited significantly more potent immune responses than id and sc administration of the vaccine (46). These observations paved the way for the clinical evaluation of PfSPZ Vaccine’s protective efficacy when administered by iv injection and, in 2013, the Sanaria team reported for the first time that five doses of 1.35 x 10^5^ iv-injected PfSPZ Vaccine (strain NF54) conferred 100% protection against an infectious challenge with PfNF54 parasites delivered by mosquito bite 3 weeks after the last immunization (47). This landmark study constituted the first demonstration that a WSp vaccine delivered by needle and syringe could confer high levels of protection against human malaria. Aided by the subsequent demonstration that PfSPZ vaccination could confer long-term protection against malaria (48), these findings laid the foundations for an ambitious plan to further the clinical development of PfSPZ Vaccine and other related products (49).

### Protection against heterologous challenge

The demonstration that five doses of the PfSPZ Vaccine could induce high levels of protection against homologous challenge in trials conducted in the USA raised several questions, including whether it would be possible to reduce the number of vaccine doses employed, and if such protection would hold upon a heterologous challenge and/or in malaria-endemic regions. The issues of dose reduction and heterologous protection were addressed in several clinical trials reported from 2017 onwards. Heterologous protection studies commonly employ the South American Pf isolate 7G8 (50, 51), which is genetically diverse from the PfSPZ Vaccine’s PfNF54 strain (52). In fact, a recent analysis of the genome, proteome and CD8^+^ T cell epitopes of various Pf strains has shown that Pf7G8 is more distant from PfNF54 than any one of more than 700 African isolates investigated, suggesting that Pf7G8 constitutes a stringent surrogate for the vaccine’s field efficacy in Africa (53). In a report from 2017, 5 doses of 2.7 × 10^5^ PfSPZ were shown to confer 92.3% and 80.0% protection against homologous (Pf3D7, a clone of PfNF54) and heterologous (Pf7G8) CHMI delivered by mosquito bite three weeks after the last immunization, respectively, but efficacy against the latter dropped dramatically to 10% twenty-four weeks after the final immunization (52). The same study also showed that a 3-dose regimen of 4.5 × 10^5^ PfSPZ conferred 86.7% and 57.1% protection against homologous CHMI by mosquito bite three and twenty-four weeks after the last immunization, respectively (52). These results indicate that heterologous protection may be less pronounced and less durable than homologous protection, raising concerns about the vaccine’s efficacy in the field. Nevertheless, another study revealed 64% protection against homologous challenge 19 weeks after the last of three immunizations with 9.0 × 10^5^ PfSPZ at 8-week intervals, and 83% of the protected subjects who underwent a repeat heterologous challenge with Pf7G8 parasites 33 weeks after the final immunization remained protected (54). Very recently, vaccination with 9 × 10^5^ PfSPZ on days 1, 8, and 29 was found to be similarly protective against homologous (PfNF54, 77% overall efficacy) and heterologous (Pf7G8, 79% overall efficacy) CHMI delivered iv at 3 or 9-10 weeks after immunization (55).

### Protective efficacy in malaria-endemic regions

The first clinical evaluation of the PfSPZ vaccine in a malaria-endemic region was conducted in healthy Malian adults, naturally exposed to malaria. Trial participants were exposed to five doses of iv-delivered 2.7 × 10^5^ PfSPZ at days 0, 28, 56, 84, and 140 during the dry season, and were actively followed up for 24 weeks during the transmission season. The results of this trial were reported in 2017 and indicated a vaccine efficacy of 51.7% (56), which is markedly lower than observed in a previous CHMI trial in the USA with a similar vaccine dose and administration schedule (52). Shortly afterwards, an identical vaccination regimen was employed to administer PfSPZ to Tanzanian adults. Challenge by homologous iv CHMI three weeks after the last immunization revealed only 20% protection, and all protected individuals remained uninfected after a re-challenge at 24 weeks (57). Interestingly, antibody responses to PfCSP in these studies, as in a PfSPZ Vaccine immunogenicity study carried out in Equatorial Guinea (58), were lower than in the volunteers in the USA (57). These observations indicate that malaria-naïve individuals in the USA respond better to the vaccine than malaria-exposed individuals in Africa. This may result from the immune modulation caused by repeated exposure to malaria, and suggests that enhancing the vaccine’s immunogenicity and achieving sterile protection in endemic regions might require increasing the dose of PfSPZ and changing the interval between immunizations (49). In an attempt to increase vaccine efficacy in Tanzania, another trial was conducted where the PfSPZ dose was increased to 9 × 10^5^ or 1.8 × 10^6^, and the number of doses was reduced to 3, at 8-week intervals. Interestingly, and perhaps somewhat surprisingly, this study revealed an association between an increase in the dose and a decrease in vaccine efficacy. In fact, while 100% of the participants who received the 9 × 10^5^ dose were protected against homologous (PfNF54) iv CHMI at 3 or 11 weeks, only 33% of those who received the 1.8 × 10^6^ dose were protected against homologous (PfNF54) iv CHMI at 7.4 weeks (59). More recently, three doses of 1.8 × 10^6^ PfSPZ at 1-, 13- and 19-week intervals afforded 51% efficacy against natural Pf transmission in Mali (60).

### Multi-dose priming

The ability to elicit effective heterologous protection is an absolute requirement for a malaria vaccine to be deployed in the field, where multiple Pf strains likely coexist. Sanaria has therefore concentrated a large part of their recent efforts on improving PfSPZ’s heterologous protection. Hypothesizing that induction of liver-resident CD8^+^ T cells, which are pivotal for vaccine efficacy (61), could be enhanced by repeated priming with low PfSPZ vaccine doses, two multi-dose priming studies followed by CHMI were recently undertaken. In a clinical trial in the USA, 5 doses of 4.5 x 10^5^ PfSPZ vaccine administered iv on days 1, 3, 5 and 7, and week 16 (referred to as multi-dose priming and delayed boosting) protected 40% of the subjects against heterologous challenge with Pf7G8 delivered by mosquito bite 12 weeks after the last immunization (62). Relevantly, in the same study, three immunizations with 9.0 × 10^5^ PfSPZ at 8-week intervals (standard dose) afforded only 20% protection against heterologous Pf7G8 challenge by mosquito bite at 12 weeks, and three 8-weekly administration of 1.8 x 10^6^ PfSPZ (escalated dose) afforded only 23% protection against heterologous Pf7G8 CHMI by mosquito bite at 24 weeks (62). More recently, the efficacy of multi-dose priming regimens of PfSPZ Vaccine against homologous (PfNF54) CHMI administered iv 6-7 weeks after the final immunization was evaluated in a clinical trial in Equatorial Guinea. In this study, four multi-dose priming regimens, with or without delayed boosting, were evaluated, all of which using doses of 9 x 10^5^ PfSPZ delivered iv: days 1, 3, 5, 7 and 113; days 1, 3, 5 and 7; days 1, 3, 5, 7 and 29; and days 1, 8, and 29. A significant 51.3% protection was only observed for the regimen in which the vaccine was administered on a 4-week schedule, on days 1, 8, and 29 (63). The delayed boosting immunization schedule yielded a protective efficacy of ~40%, which is similar to that observed in the USA trial (62), but was not statistically significant (63). Perhaps surprisingly, protection afforded by the 2-dose multi-prime regimen (days 1, 8, and 29; 51.3%) was higher than that afforded by 4-dose multi-prime (days 1, 3, 5, 7 and 29; 10.7%), clearly a matter that demands additional investigation.

### Vaccination of children and infants

Malaria exerts its heavier mortality burden on children and infants, with 77% of total malaria deaths in 2020 occurring under the age of 5 years-old (64). With this in mind, Sanaria, Inc. initiated an assessment of the safety and feasibility of iv administration of the PfSPZ Vaccine, aiming to conduct an efficacy trial on this age group. These assessments took place in Tanzania (65) and Kenya (66, 67), and were accompanied by a careful analysis of caregiver and community perceptions and experiences regarding participation in these studies (68). These efforts culminated in a recently reported phase 2 trial conducted in western Kenya on a population of 336 infants aged 5-12 months, naturally exposed to malaria. The vaccine was administered in three iv doses of 4.5 × 10^5^, 9.0 × 10^5^ or 1.8 × 10^6^ PfSPZ spaced by 8 weeks, with a 12-month follow-up period. Although vaccine efficacy against clinical malaria was estimated at 45.8% in the highest-dose group at the study’s 3-months exploratory endpoint, significant protection against Pf infection was not observed in any dose group at the 6 months primary endpoint (69). These disappointing findings indicate that immune responses to the PfSPZ Vaccine are age-dependent, and may be explained by major differences between infants and older children and adults in the priming of PfSPZ-specific T cell responses (65, 69), and/or by the presence of low-level Pf parasitemia at the time of administration of the first vaccine dose (69, 70). In any case, these results clearly do not support the use of the PfSPZ Vaccine in infant populations, whose immune systems are immature, particularly for T-cell responses (71).

## Genetically-attenuated parasites

*Plasmodium* parasites express several genes encoding pre-erythrocytic stage-specific proteins, some of which may be essential for the parasite’s intra-hepatic development (72). Genetically-attenuated parasites (GAP) have been engineered to abrogate the expression of one or more genes essential for completion of their developmental process in the liver. Targeted deletion of these genes results in parasites that are able to infect hepatocytes but arrest their liver-stage development at defined points, remaining unable to establish a symptomatic blood-stage infection *in vivo* (73). A potential advantage of GAP- over RAS-based immunization is that the former constitute a homogeneous population of parasites with defined genetic identity and attenuation phenotype, which may be designed to induce optimal protective immunity (74). It is usually accepted that the immunity induced by parasites whose liver development arrests later is superior to that induced by early-arresting parasites (75, 76). Therefore, the development of a late-arresting PfGAP that can elicit effective protective immunity against malaria remains an attractive objective to which much attention has been devoted.

### GAPs: From mice to humans

Effective vaccination employing genetically attenuated *Plasmodium* parasites was first demonstrated in rodent models of malaria in the mid-2000’s. In 2005, Mueller *at al.* and van Dijk *et al.* showed that immunization of mice with Pb sporozoites deficient in the upregulated in infective sporozoites gene 3 (*uis3*) or in the *p36p* gene, respectively conferred complete protection against a challenge with infectious Pb sporozoites (77, 78). Over the next few years following these landmark studies, several reports emerged showing that highly effective protective immunity could be elicited by immunization by iv injection of other rodent parasite mutants, including *p52*-/*p36*-deficient *P. yoelii* (Py) (79), *uis3*-/*uis4*-deficient Pb (80) and Py (81), and purine nucleoside phosphorylase (*pnp*)-deficient Py (82), multidrug resistance-associated protein 2 (*mrp2*)-deficient Pb (83), and *b9*-/*slarp*-deficient Pb, followed by an iv challenge employing fully infective sporozoites of the same species (84).

Naturally, the success for GAP-based vaccination in rodents sparked an interest in the use of this approach to create vaccine candidates against human malaria. The genetic design of replication-competent vaccine strains holds the promise for a potent, broadly protective malaria vaccine (85). The development of appropriate genetic manipulation methods enabled the targeted deletion of genes in order to create Pf GAPs that arrest during hepatic development and that lack drug-resistance markers (86–88). Subsequent technical advances in genetic manipulation enhanced the efficiency and pace for generation of transgenic *Plasmodium* parasites (85). The first Pf GAP was reported in 2009 and consisted of a Pf parasite lacking the *p52* and *p36* genes, whose liver arrest was confirmed *in vitro* and in a liver-humanized mouse model (87). Since then, several other Pf GAPs have been reported in the literature, including Pf*b9* ^−^/*slarp* ^−^ (84), Pf*p52* ^−^/*p36* ^−^/*sap1* ^−^ (89) and Pf*mei2* ^−^ (90). Several of these candidates have been, are currently, or will likely undergo evaluation in a clinical setting.

### Clinical evaluation of Pf GAP vaccines

The number of Pf GAP candidates tested in humans is currently limited. The first report of such a clinical study dates from 2013, when Pf*p52* ^−^/*p36* ^−^ sporozoites (87) were administered to six malaria-naïve volunteers by the bites of infected female *Anopheles stephensi* mosquitoes. Subjects were initially exposed to 5 bites/volunteer, which was followed by exposure to ~200 bites/volunteer one month later. Although all volunteers remained blood stage-negative after the low dose exposure, one volunteer developed parasitemia after exposure to 263 bites, activating a Stopping Rule in the study (91). Genotyping analysis confirmed that the parasite in the peripheral circulation of this volunteer was Pf*p52* ^−^/*p36* ^−^, showing that a breakthrough infection, rather than a reversion to wild-type Pf, had occurred (91). This observation highlights the need to identify gene deletions, or a combination thereof, that ensure the parasite’s complete arrest in the liver of the immunized subjects. In an attempt to achieve this, an additional deletion was included to generate the Pf*p52* ^−^/*p36* ^−^/*sap1* ^−^ parasite, termed PfGAP3KO (89). To confirm immunization safety, PfGAP3KO was administered to 10 subjects by a single exposure to the bites of 150 to 200 bites per volunteer. All participants in this study remained blood stage-negative, indicating complete attenuation of PfGAP3KO in humans, and paving the way for the evaluation of its protective efficacy in the clinic (89).

The first Pf GAP to have undergone an evaluation of its protective efficacy in humans is Pf*b9* ^−^/*slarp* ^−^ (84). Aseptic, purified, and cryopreserved Pf*b9* ^−^/*slarp* ^−^ sporozoites were manufactured by Sanaria, Inc., creating the PfSPZ-GA1 Vaccine. No breakthrough infections were observed following the iv administration of three doses of 4.5 × 10^5^ or 9.0 × 10^5^ PfSPZ-GA1 Vaccine at 8-week intervals (92). Subjects were then challenged by mosquito bite CHMI with PfNF54 parasites 3 weeks after the last immunization. Although all vaccine groups showed a significant increase in pre-patency time, only 1 of 12 volunteers in the 4.5 × 10^5^ PfSPZ-GA1 group and 2 of 13 volunteers in the 9.0 × 10^5^ PfSPZ-GA1 group were sterily protected (92). Even though these results may appear somewhat disappointing, this is a landmark trial in that it constitutes the first clinical assessment of the protective efficacy of a Pf GAP vaccine. Furthermore, it should be noted that all volunteers from a Pf RAS control group, immunized with three doses of 4.5 × 10^5^ PfSPZ Vaccine, developed parasitemia (92), which may reflect a particularly high stringency of the PfNF54 mosquito bite challenge employed in this study.

The clinical evaluation of PfGAP3KO’s immunogenicity and protective efficacy was reported very recently. In this trial, the vaccine was delivered by three (with 4 weeks between the first and second vaccinations and the 8 weeks between the second and third vaccinations) or five (with 4 weeks between the first four vaccinations and 8 weeks between the fourth and fifth vaccinations) immunizations, with ~200 PfGAP3KO-infected mosquito bites per immunization. CHMI was carried out by the bites of PfNF54-infected mosquitoes either 4 weeks after the last immunization of the 6 volunteers in each of study arms 1 and 2, or 26 weeks after the first CHMI for study participants in both study arms who did not have any detectable Pf infection after the first CHMI. The vaccine protected 50% of the volunteers in either study arm after the first CHMI, and protected 1 of the 6 volunteers who undertook the second CHMI (93).

The road ahead for Pf GAP vaccination remains wide open, with efforts ongoing towards the identification of late-arresting replication-competent Pf parasites that are completely attenuated and highly immunogenic. Moreover, existing mutants, such as Pf*mei2* ^−^, are already undergoing clinical evaluation, and several others are likely to follow. Finally, the possibility of iteratively improving these parasites through the expression of additional antigens or immunomodulatory elements offers the prospect of a rationale for the creation of increasingly efficacious and versatile Pf GAP candidates (85).

## Chemoprophylaxis and sporozoites

Depending on their molecular target and mode of action, antiplasmodial drugs may act either on multiple or only on specific stages of the parasite’s life cycle. Immunization by ChemoProphylaxis and Sporozoites (CPS) relies on the ability of an antiplasmodial compound to provide a prophylactic cover against the symptomatic stage of *Plasmodium* infection following the administration of non-attenuated sporozoites. Thus, the inoculated, replication-competent, parasites are able to infect, develop and egress from hepatic cells unencumbered, but are eliminated prior to egress or following merozoite release into the blood stream, during the first wave of invasion of red blood cells (94). Liver infection elicits potent pre-erythrocytic immune responses, while the appearance of disease symptoms is prevented by the presence of the circulating drug. Unrestricted liver stage growth expands parasite biomass and antigenic repertoire to a greater extent than what occurs with RAS and GAP, potentially enhancing immunogenicity and decreasing the dose of immunizing parasites required for protection. In addition, the presence of an abortive blood-stage infection may elicit humoral immune responses against blood-stage *Plasmodium* antigens (95). Early pre-clinical studies showed that immunization of mice with fully infectious Pb (96, 97) or Py (98) sporozoites under chloroquine treatment, a drug that specifically targets blood stage parasites (99), conferred significant protection against a sporozoite challenge with the same parasite species. Since then, similar results have been obtained employing other antiplasmodial drugs, such as primaquine (100), mefloquine (101), pyrimethamine (102), piperaquine (103), artesunate (104), clindamycin (105), azithromycin (105) and arteether (106). More recently, CPS employing *P. knowlesi* (Pk) sporozoites and chloroquine was also shown to confer significant protection against Pk infection in a non-human primate model (107). Collectively, these pre-clinical observations paved the way to a wide array of studies aimed at assessing the potential of CPS immunization for vaccination against human malaria.

### CPS immunization by mosquito bite

The first-in-humans demonstration that CPS immunization could afford high levels of sterile protection against Pf infection was provided by a landmark study in 2009, carried out at Nijmegen’s Radboud University Medical Centre. In this seminal study, ten volunteers were exposed to the bites of 12 to 15 PfNF54-infected mosquitoes in three immunization sessions at 1-month intervals, whilst under the cover of a prophylactic chloroquine regimen. Five control subjects received an equivalent number of non-infected mosquito bites and were subjected to a similar chloroquine regimen. Both groups of volunteers were challenged by homologous CHMI delivered by mosquito bite 8 weeks after the last immunization dose (4 weeks after the discontinuation of chloroquine prophylaxis). Whereas all control subjects developed PfNF54 parasitemia, all immunized volunteers were protected against infection, indicating a striking 100% homologous protective efficacy of this immunization method (108). Importantly, a homologous re-challenge of six protected volunteers 2.5 years after the original study revealed that four of them remained sterilely protected, while the remaining two showed prolonged prepatent periods (109).

In a trial aimed at discerning the contributions of pre-erythrocytic and erythrocytic immunity for the protection afforded by Pf CPS vaccination, 4 out of 5 subjects (80%) taking chloroquine prophylaxis and immunized by 3 exposures to the bites of 15 PfNF54-infected mosquitoes at 1-month intervals were fully protected against a homologous CHMI by mosquito bite (110). In another group of 9 similarly immunized volunteers, none was protected against a blood-stage challenge by iv administration of asexual PfNF54 parasites, showing that protection against malaria CPS immunization is entirely mediated by pre-erythrocytic immunity (110). In a subsequent trial, 60 and 70% homologous protection was observed for volunteers under either chloroquine or mefloquine prophylaxis, respectively, who were exposed 3 times to 8 PfNF54-infected mosquitoes at monthly intervals (111).

The enthusiasm generated by the high protective efficacy observed in these homologous CHMI trials led to the assessment of the protection conferred by CPS immunization against heterologous parasite strains. Thus, in a follow-up study, 16 volunteers previously immunized by CPS employing PfNF54 parasites delivered by mosquito bite and homologously challenged with the same parasite strain were re-challenged 14 months after the last immunization with Pf strain NF135.C10. Only 2 out of 13 volunteers that were previously fully protected against PfNF54 were also fully protected against Pf NF135.C10, while the remaining 11 displayed an increased pre-patent period (112). These somewhat disappointing results were made even more so by the results of a subsequent clinical trial. There, CPS immunization with PfNF54 protected 5 out of 5 volunteers against a PfNF54 challenge 14 weeks after the last immunization, but sterilely protected only 2 out of 10 and 1 out of 9 volunteers against CHMI with Pf strains NF135.C10 and NF166.C8, respectively, all delivered by mosquito bite (113). These findings raise important questions regarding the potency of the immune responses required for effective heterologous protection following CPS immunization and the optimization thereof. This may involve the use of an immunizing Pf strain with intrinsically higher liver stage infectivity, an increase in the immunization dose or an alteration of the immunization regimen (113). Some of these challenges can at least be partially addressed by resorting to iv administration of the immunizing parasites, as discussed below.

### Enter Sanaria’s PfSPZ-CVac

In view of the promising results of early CPS vaccine trials in the clinic, the team at Sanaria, Inc. and its collaborators posited that PfSPZ Challenge could serve as a replacement for mosquito bite delivery of immunizing Pf parasites, hence giving rise to a CPS vaccine approach termed PfSPZ- Chemoprophylaxis Vaccine (PfSPZ-CVac) (49). In the first clinical trial with PfSPZ-CVac, 3-4 id administrations of 7.5 x 10^4^ PfSPZ employing chloroquine as the drug partner induced no sterile protection against homologous CHMI with PfSPZ Challenge (114). With the benefit of hindsight, it is now clear that this is not a surprising result, given the poor immunogenicity of vaccine administration by the id route, as observed in PfSPZ vaccine studies ongoing at the time (46, 47). Thus, in a subsequent landmark trial carried out at the University of Tübingen, PfSPZ-CVac was administered iv, with chloroquine as the partner drug. A dose-dependent protective effect of the vaccine was observed, with 100% of the volunteers immunized by three doses of 5.12 × 10^4^ sporozoites at 28-day intervals being protected against homologous iv CHMI with PfSPZ Challenge (PfNF54) 10 weeks after the last immunization (115). Remarkably, not only was this the first time that complete sterile immunity by PfSPZ-CVac was observed in the clinic, but also this was achieved with sporozoite doses 1-2 orders of magnitude lower than those required by RAS immunization with PfSPZ Vaccine, as outlined above. These results confirmed the high immunogenicity of the PfSPZ-CVac immunization approach, opening the door for further optimization of the immunization regimen and its assessment against heterologous CHMI or in the field (116).

The first assessment of PfSPZ-CVac in a malaria-endemic region took place in Equatorial Guinea and was reported in 2021. In this clinical trial, 3 doses of 2.7 × 10^6^ PfSPZ Vaccine or 1.0 × 10^5^ PfSPZ-CVac were administered at 8- or 4-week intervals, respectively, to different groups of volunteers. Immunized subjects underwent homologous CHMI by iv administration of PfSPZ Challenge (PfNF54) at a median of 14 weeks after the last immunization. Vaccine efficacies were 27 and 55% for PfSPZ Vaccine and PfSPZ-CVac, respectively, and were not statistically different from each other (117). Pre-patency as assessed by thick blood smear was significantly longer for PfSPZ Vaccine, but not for PfSPZ-CVac recipients, than controls (117). This trial constitutes the first head-to-head comparison of PfSPZ Vaccine and PfSPZ-CVac efficacies. It should be noted that the efficacy of both immunizations was lower than that observed in homologous CHMI studies in malaria-naïve volunteers employing lower vaccination doses (54, 115), once again indicating that immunization regimens in the field require further optimization.

### Heterologous protection by PfSPZ-CVac vaccination

The issue of vaccination dose began to be assessed in a trial reported in 2021, where PfSPZ-CVac was used in combination with either chloroquine or pyrimethamine at a dose of 2 × 10^5^ sporozoites, a 4-fold increase relative to that employed in the Mordmuller *et al.* study (115). In this study, subjects received 3 monthly immunizations with either partner drug, and underwent CHMI by iv administration of PfSPZ Challenge 3 months after the last immunization. The data revealed 100% heterologous (Pf7G8) protection in the chloroquine group, whereas 87.5 and ~78% protective efficacy was observed against homologous (PfNF54) and heterologous (Pf7G8) challenge, respectively, in the pyrimethamine group (118). These remarkable results constitute the first demonstration that high levels of heterologous protection can be achieved for at least 3 months through PfSPZ-CVac vaccination, which is significantly higher than what had been observed for RAS immunization with 9 × 10^5^ PfSPZ Vaccine (62). However, in a very recent study in Mali, 3 doses of 2 × 10^5^ PfSPZ-CVac (chloroquine) administered at 0, 4 and 8 weeks afforded only an estimated, non-statistically significant, protective efficacy of ~33% against naturally transmitted Pf infection over a 48-week surveillance period spanning wet and dry seasons (119).

### Condensed PfSPZ-CVac immunization regimens

Also in 2021, a condensed immunization regimen employing PfSPZ-CVac and chloroquine was attempted for the first time. Inoculation of 1.1 × 10^5^ sporozoites, twice the dose employed in the Mordmuller *et al.* study (115), on days 1, 6 and 29, yielded 77% protection against heterologous (Pf7G8) iv CHMI with PfSPZ Challenge 12 weeks after the last immunization (120). The importance of this study lies not only on the high protective efficacy observed, but also on the fact that in the immunization regimen employed chloroquine was administered only on the days of vaccine inoculation, limiting to three the number of visits to complete vaccination (120). In yet another study from 2021, two condensed regimens of three administrations of 5.12x10^4^ PfSPZ-CVac seven days apart and of 1.024x10^5^ PfSPZ-CVac five days apart, using chloroquine as the partner drug, were assessed in the clinic. The two regimens gave very different protections against homologous CHMI with PfSPZ Challenge (PfNF54), with the 7-day group showing 0% protective efficacy, and the higher-dose, 5-day group displaying 75% protective efficacy (121). It should be noted that vaccine administration to the former group coincided with patent parasitemia, suggesting that this may be associated with the observed lack of sterile immunity (121). Finally, in a very recent assessment of accelerated PfSPZ-CVac vaccination regimens, volunteers underwent three-dose immunization regimens at days 0/14/28 or at days 0/5/10, employing 5.12 × 10^4^ sporozoites/dose and chloroquine as the partner drug. Homologous CHMI was performed by iv administration of PfSPZ Challenge (PfNF54) 10 weeks after the last immunization. The two immunization regimens yielded similar protective efficacies of 67 and 63% for 28- and 10-day vaccination schedules, respectively, but the latter resulted in more pronounced cellular and humoral immune responses than the former (122). Collectively, these results pave the way for further development of an effective condensed regimen of PfSPZ-CVac immunization, capable of eliciting protective immunity in the field.

## *P. berghei*-based vaccination against human malaria

Rodent *Plasmodium* parasites are the most widely employed models of malaria research, particularly in what concerns the investigation of the pre-erythrocytic stages of infection (123). In recent years, rodent malaria parasites have also emerged as potential candidates for WSp immunization against human malaria. The idea draws from the origins of vaccination, when Edward Jenner unknowingly established the notion of cross-species protective immunity, by successfully using cowpox to vaccinate humans against smallpox (124). The notion that a similar principle may apply to Pb and human malaria parasites is supported by the presence of cross-species epitopes in different malaria parasites (125), and is strengthened by the high percentage of predicted T cell epitopes shared between the former and the latter (126). Besides, Pb’s high amenability to genetic modification, solidified by years of experience in this area, enables the insertion of selected human *Plasmodium* antigens into neutral loci of its genome, effectively turning the rodent parasite into a unique platform for expression of heterologous *Plasmodium* antigens (127). Immunization with such chimeric Pb sporozoites is therefore expected to elicit not only cross-species immune responses, but also targeted immunity against human malaria parasites arising from those heterologous immunogens (128).

### Pre-clinical validation of Pb-based WSp vaccination

The concept of Pb-based WSp vaccination was validated in 2018 through the generation of PbVac, a Pb parasite that expresses PfCSP under the control of the strictly pre-erythrocytic Pb*uis4* promoter (126). Pre-clinical characterization of PbVac showed that it expresses both the endogenous PbCSP and the heterologous PfCSP at the surface of sporozoites and liver stages, and that it displays wild-type Pb-like mosquito and hepatic infectivity levels (126). Employing liver- and blood-humanized mouse models, PbVac was also shown to invade and develop inside human hepatocytes and to be unable to replicate inside human erythrocytes. Moreover, and crucially, PbVac was found to infect human primary hepatocytes with significantly higher efficacy than Pf, which may potentially entail high levels of human liver infectivity (126). Immunization of rabbits by the bites of PbVac-infected mosquitoes elicited cross-species cellular immune responses, as well as PfCSP-specific antibody responses that functionally inhibit infection of human hepatocytes by Pf, both *in vitro* and in liver-humanized mice (126). Collectively, these data unequivocally demonstrated PbVac’s potential for immunization against Pf malaria, warranting its evaluation in the clinic. However, this posed a significant challenge, not only because there was no previous history of experimental administration of rodent malaria parasites to humans, but also due to the fact that PbVac is a genetically modified organism, and that sporozoites of this parasite can only be generated in mosquitoes infected by feeding on the blood of infected mice. Thus, several additional studies were performed to ensure the safety of PbVac for human use, including the creation of a Master Cell Bank, whole-genome sequencing of the transgenic parasite, a complete set of microbiological analyses, and tissue distribution and drug-sensitivity studies (129). The complete set of pre-clinical data gathered in these studies (126, 129) paved the way for its assessment in humans.

### Clinical assessment of PbVac

The first-in-humans assessment of PbVac was reported in 2020 and consisted of a phase 1/2a clinical trial, in which PbVac sporozoites were administered to volunteers by the bites of infected female *A. stephensi* mosquitoes. Safety was assessed in a phase 1 dose-escalation study, in which groups of volunteers were exposed to the bites of 5, 25 and 75 PbVac-infected mosquitoes, with no breakthrough infections or serious adverse events recorded (130). In phase 2a of the study, 12 volunteers were immunized by four exposures to the bites of 75 PbVac-infected mosquitoes, spaced by 4 (between the first and second and between the second and third immunizations) or 8 (between the third and fourth immunizations) weeks, and were challenged 3 weeks after the last immunization by PfNF54-infected mosquito bites. A significant delay in blood stage patency and a significantly lower parasite density at first detection in the blood was observed in immunized volunteers, corresponding to an estimated 95% decrease in PfNF54 liver load for vaccinated subjects relative to non-immunized controls (130). It should be noted that the 4 x 75 PbVac-infected mosquito bites employed in this study corresponds to a much lower vaccine dose than that delivered by the more than 1000 mosquito bites previously used for immunization with Pf RAS (11). Thus, although no sterile protection was observed in the PbVac study, the marked reduction in liver parasite load triggered by immunization with the clearly sub-optimal dose employed, alongside the dose-dependent humoral and cellular immune responses observed (130), support further exploration of Pb-based vaccination against malaria. To this end, the production of aseptically purified, vialed, cryopreserved PbVac sporozoites that can be administered by parenteral injection at defined doses is currently ongoing in collaboration with Sanaria, Inc. Furthermore, the possibility of inserting multiple heterologous genes in the Pb genome (131) under the control of suitable pre-erythrocytic promoters (132) enables the generation of transgenic Pb parasites that express genes from different human *Plasmodium* parasites and from different stages of their life cycle. This possibility is particularly appealing in the case of Pv, for which an *in vitro* culture system is yet to be achieved (133), which severely limits the development of a WSp vaccine. Thus, transgenic Pb parasites expressing suitable Pv antigens may serve as unique surrogates for WSp vaccination against this human malaria parasite.

## Immune responses elicited by whole-sporozoite vaccination

WSp vaccines primarily aim at boosting the host’s immunity through the generation of effective and long-lasting immune responses that control and/or eliminate the parasite during the pre-erythrocytic stage of its life cycle. The investigation of these immunological mechanisms and their correlation with protection have been the focus of multiple studies that led to a thorough, yet still incomplete, picture of the immunity that ensues following vaccination, as recently reviewed (134–136). Although several studies have suggested a relation between some immune parameters and protection, a definitive immune correlate of protective efficacy of WSp vaccination remains to be clearly identified. Studies in mice and non-human primates have provided robust evidence that a large part of the pre-erythrocytic immune response that leads to protection is cell-mediated in the liver [reviewed in (61, 137)]. However, the fact that, in humans, immune parameters can only be analyzed in the peripheral circulation constitutes a limitation to the assessment of the global WSp-associated immunological landscape in the clinic. Moreover, it is likely that different WSp vaccines may produce distinct humoral and cellular response signatures that define protective immunity. In this chapter we will outline the main humoral and cellular immune responses identified during the clinical assessment of a variety of WSp vaccine candidates.

### Antibody-mediated responses

Vaccines commonly act by inducing an antibody-mediated response against specific microorganisms or their constituents. The humoral responses induced by WSp vaccines are largely directed at pre-erythrocytic antigens, with CSP, the most abundant protein on the surface of sporozoites, representing the hallmark parasite target [reviewed in (134, 136, 138, 139)]. Sporozoite- or CSP-specific antibodies are consistently induced in response to WSp vaccination of malaria-naïve individuals, and some studies have reported a correlation of antibody titers with RAS (47, 69), CPS (115), GAP (92) or PbVac (130) immunization doses, or with PfSPZ-induced protection (48, 92). Importantly, pre-exposure has been identified as a limiting factor for the magnitude of the humoral responses elicited by RAS (57, 58, 140) and PfSPZ-CVac (117) immunizations. Whether a similar effect is observed following immunization with other types of WSp vaccines remains to be addressed.

In addition to the magnitude of the humoral response, it is also important to assess the functionality of the antibodies generated by vaccination. Antibodies against sporozoites or their antigens may limit the infection in several ways, including by decreasing their motility (141), inhibiting hepatocyte invasion and parasite development (142), or mediating their destruction through mechanisms such as antibody-dependent cytotoxicity or phagocytosis upon opsonization (143, 144). The functionality of the circulating antibodies induced by WSp vaccination can be assessed by a variety of *in vitro* assays or *in vivo* studies, as recently reviewed (145). An important role for antibodies in pre-erythrocytic immunity was initially established through the observation that patency following administration of Pb sporozoites to naïve mice was delayed by passive transfer of serum from RAS-immunized mice (146). A functional role for antibodies elicited by PfSPZ (48), CPS (147) and Pf GAP3KO (89) immunization has been demonstrated *in vivo* using liver-humanized mouse models.

Different WSp vaccine approaches lead to distinct extents of parasite development in the liver, hence differing in the breadth of *Plasmodium* antigens presented to the host. Accordingly, antibodies to asexual and sexual erythrocytic antigens were low to undetectable following PfSPZ Vaccine (47) and early-arresting GAP (91) immunizations, while humoral responses against both pre-erythrocytic and cross-stage *Plasmodium* antigens are induced by CPS vaccination (148). Functional antibodies against the immunodominant CSP, which is common across WSp vaccine strategies, are prevalent in all WSp immunization approaches [reviewed in (134); see also (92, 130, 149–151)]. Nonetheless, antibodies against non-CSP proteins from CPS-immunized volunteers were shown to block Pf parasite development in hepatocytes *in vitro* and *in vivo* (152). In fact, several other antigens besides CSP currently constitute promising vaccine candidates, including thrombospondin-related adhesion protein (TRAP) (153) and cell-traversal protein for *Plasmodium* ookinetes and sporozoites (CelTOS) (154). Excitingly, Pb-based WSp vaccination (126, 130) offers a platform that may be used as a backbone for insertion of multiple genes, to elicit tailored humoral immune responses that enhance and/or synergize with those induced against CSP. This strategy may trigger humoral immunity against multiple Pf strains, as well as against other *Plasmodium* species, such as Pv, to overcome current limitations of the existing WSp vaccination approaches.

### Cellular immunity

Cellular immunity is critical for the protection elicited by RAS immunization in rodent and non-human primate models, and most pre-clinical data indicate a central role for CD8^+^ T cells and interferon-γ (IFN-γ) in protection by this vaccination approach (46, 155, 156). In addition, other cell populations, including CD4^+^ T cells, γδ T cells and natural killer (NK) cells, can also play a role in mediating protection [reviewed in (135, 137)].

CD8^+^ T cells recognize pathogen-derived peptides bound to MHC class I molecules on the surface of antigen presenting cells or infected cells, and can eliminate liver stage *Plasmodium* parasites either directly, such as through perforin-mediated lysis (157), or indirectly, through cytokine (e.g. IFN-γ, TNF-α) production [reviewed in (135, 137)]. Sterile immunity induced by RAS vaccination in mice is abolished upon depletion of CD8^+^ T cells or IFN-γ (155, 158), and IFN-γ directly impairs *Plasmodium* development in human hepatocytes in culture (159). In general, immunizations of humans by RAS (48, 65, 69) and CPS (92, 115) do not consistently nor robustly induce Pf-specific CD8^+^ T cells in the blood of vaccinated subjects. Nevertheless, some studies reported the detection and dose-dependent increase in the frequency of those cells after vaccination by RAS (47), GAP (91, 92) or PbVac (130), although this did not correlate with protection or patency. Moreover, increased granzyme B expression by CD8^+^ T cells was associated with protection following CPS vaccination (111). The overall suboptimal detection of parasite-specific CD8^+^ T cells in the blood is likely associated with their predominant tissue residency. Indeed, Pf-specific IFN-γ-producing CD8^+^ T cells produced upon RAS immunization of non-human primates are mainly localized to the liver, where they can be present at up to 100 times higher frequencies than in the blood (46, 48). These studies have highlighted the importance of vaccine administration route (iv>>id or sc), dose and schedule on the formation of tissue-resident CD8^+^ T cell responses, which likely extends to the other WSp immunization strategies.

CD4^+^ T cells can have a multiplicity of roles in mediating protective immunity in malaria, including aiding the survival and differentiation of CD8^+^ T cells (160, 161), the development of efficient B cell responses (162, 163), or by acting directly through pro-inflammatory cytokine (eg. IFN-γ, TNF-α, IL-2) production (reviewed in (135, 137, 164). Many studies have reported the presence of Pf-specific CD4^+^ T cells, and particularly of polyfunctional memory Th1 cells (producing IFN-γ, TNF-α and/or IL-2), in the blood of volunteers immunized with RAS (47, 48, 54, 57, 65), CPS (92, 115), GAP (91, 92) and PbVac (130), but they were only associated with protective immunity following CPS vaccination (108, 109, 115). In addition, the increased expression of the degranulation marker CD107a on CD4^+^ T cells has also been associated with protection against homologous (111) but not heterologous (113) challenge following CPS immunization. Importantly, Pf-specific polyfunctional memory CD4^+^ T cell responses were low to undetectable in PfSPZ-vaccinated infants in Tanzania (65) and Western Kenya (69), raising concerns regarding the implementation of the PfSPZ vaccination strategy in this immunologically immature population.

γδ T cells, which represent 2-5% of total T cells in humans, are unconventional T cells that are not restricted by classical MHC-mediated antigen presentation. The major subset of γδ T cells in the blood, Vγ9^+^Vδ2^+^, recognizes stress or pathogen-related phosphoantigens that specifically and robustly activate them to proliferate, secrete cytokines (such as IFN-γ and TNF-α), and display cytotoxic behavior [reviewed in (165, 166)]. Accordingly, human γδ T cells are innate responders to *Plasmodium* parasites *in vitro* (167) and are able to directly kill merozoites (168). Data from animal studies have provided evidence that γδ T cells can inhibit *Plasmodium* hepatic development (169), and are necessary for the generation of protective CD8^+^ T cell responses and for sterile protection following RAS vaccination (170), among other functions (reviewed in (135, 137, 171). In RAS vaccine clinical trials, γδ T cells expanded upon immunization of malaria-naïve and pre-exposed volunteers (47, 48, 54, 170), and the frequency of Vδ2^+^ γδ T cells was found to be predictive of protection, both at baseline and prior to CHMI (48). Vδ2^+^ γδ T cell expansion was further reported in some studies following CPS (115, 118) and PbVac (130) immunization. Hence, γδ T cells, and specifically the Vδ2^+^ subset, represent a potential correlate of protection that warrants further exploration (136).

NK and NKT cells are important innate and innate-like effector cells that are abundant in the liver, and have been implicated in cell-mediated immunity to liver stage *Plasmodium* infection [reviewed in (172, 173)]. Although not extensively analyzed in the context of WSp vaccination, NK and NKT cells were shown to contribute to the increase in IFNγ production by lymphocytes responding to Pf following CHMI (174), and NK cells were found to upregulate activation and proliferation markers during CPS immunization (94). Importantly, an increase in NK and NKT cell frequencies was found following PbVac immunization, which, for the latter population, correlated the prepatent period of vaccinated individuals (130). Future vaccine development studies should further investigate in depth these and other innate and innate-like populations, as well as related pathways, in light of recent data on their involvement in immune signatures that potentially correlate with protection (175).

## Final remarks: Lessons from the past, challenges for the future

Looking back to the history of research on WSp vaccines against malaria, it is clear that much has been achieved, particularly during this last decade. While until the early 2010’s progress was relatively slow, and only a handful of clinical trials had been performed, this number has risen dramatically since then. During this period, Sanaria Inc.’s achievements have revolutionized the field, transforming an attractive, yet unpractical, immunization strategy into a family of injectable products suitable for vaccination and CHMI. The PfSPZ Vaccine alone has now been administered to more than 1700 volunteers in over 20 clinical trials, PfSPZ-CVac has been assessed in a large array of clinical studies and immunization regimens, and PfSPZ Challenge has been used for CHMI of several dozen subjects (31). It was also during this period that Pf GAP vaccination was first evaluated in the clinic, as was a novel WSp immunization strategy based on the use of genetically modified Pb parasites. We presently understand the elicitation of immunity by WSp vaccines better than ever before, and major technical hurdles that once seemed unsurmountable have now been overcome. And yet, the road travelled so far was not without pitfalls, and many important challenges still lay on the path ahead. Despite progress in the automation of mosquito dissections (176), an effective system for *in vitro* production of Pf sporozoites remains unavailable. Nevertheless, Sanaria, Inc. have publicly announced that major achievements have been made in this regard, and it is very likely that these findings will be published in the near future. Although much has been learned from the immunological analyses of clinical samples from participants in multiple trials (145), immune correlates of malaria vaccine efficacy remain largely undefined (136). On the other hand, the disappointing results of the only clinical trial of a WSp vaccine in infants raises justified concerns about the effectiveness of this immunization approach in that age group (69). Moreover, the higher protective efficacy in malaria naïve volunteers when compared to malaria pre-exposed volunteers (47, 52, 56, 59), as well as the variable levels of protection afforded by different regimens of PfSPZ-CVac vaccination (115, 118, 120, 121), suggest that additional optimization of immunization regimens with these vaccines is required. Finally, the relatively low clinical efficacy of the PfSPZ-GA1 and PbVac candidates (92, 130) demands additional development of these promising, yet still suboptimal, vaccination approaches. Several of these issues will more than likely be addressed in future clinical trials, either planned or ongoing. According to clinicaltrials.gov, there are currently several active, recruiting, or not yet recruiting trials of WSp malaria vaccines, including studies aimed at assessing PfSPZ Vaccine efficacy in Malian women of childbearing age (NCT03989102) and in Malian children (NCT04940130), as well as against heterologous CHMI in malaria-naïve USA adults (NCT04966871), and a head-to-head comparison between an early-arresting [GA1: Pf*b9* ^−^/*slarp* ^−^ (84)] and a late-arresting [GA2: Pf*mei2* ^−^ (90)] GAP is currently ongoing at the Leiden University Medical Center (NCT04577066). Plans are also being made for the clinical evaluation of the safety and protective efficacy of parentally injected PbVac. Moving forward, these and other studies will continue to compound our accumulated knowledge on human immunization with WSp malaria vaccines, bringing their use for preventing disease and, ultimately, contributing to its elimination, ever closer to reality.

## Author contributions

All authors listed have made a substantial, direct, and intellectual contribution to the work and approved it for publication.

## Funding

MP acknowledges the “la Caixa” Foundation for Grant HR21-848, the GSK OpenLab Foundation for grant TC269, and Fundação para a Ciência e Tecnologia (FCT) for grant PTDC-SAU-INF-29550-2017. HN-C acknowledges funding from FCT (reference DL57/2016/CP1451/CT0011).

## Acknowledgments

Helena Pinheiro is gratefully acknowledged for designing **Figure 1** of the manuscript.

## Conflict of interest

The authors declare that the research was conducted in the absence of any commercial or financial relationships that could be construed as a potential conflict of interest.

## Publisher’s note

All claims expressed in this article are solely those of the authors and do not necessarily represent those of their affiliated organizations, or those of the publisher, the editors and the reviewers. Any product that may be evaluated in this article, or claim that may be made by its manufacturer, is not guaranteed or endorsed by the publisher.
